# Long-Term Follow-Up of Health-Related Quality of Life and Short-Term Intervention with CFTR Modulator Therapy in Adults with Cystic Fibrosis: Evaluation of Changes over Several Years with or without 33 Weeks of CFTR Modulator Therapy

**DOI:** 10.3390/healthcare11212873

**Published:** 2023-10-31

**Authors:** Wolfgang Gruber, Matthias Welsner, Christopher Blosch, Stefanie Dillenhoefer, Margarete Olivier, Folke Brinkmann, Cordula Koerner-Rettberg, Sivagurunathan Sutharsan, Uwe Mellies, Christian Taube, Florian Stehling

**Affiliations:** 1 Pediatric Pulmonology and Sleep Medicine, Cystic Fibrosis Center, Children’s Hospital, University of Duisburg-Essen, 45147 Essen, Germany; 2 Institute of Human Nutrition and Food Science, Christian-Albrechts-Universität zu Kiel, 24118 Kiel, Germany; 3 Department of Pulmonary Medicine, University Hospital Essen–Ruhrlandklinik, Adult Cystic Fibrosis Center, University of Duisburg-Essen, 45239 Essen, Germany; 4 Department of Pediatric Pulmonology, University Children’s Hospital, Ruhr University Bochum, 44801 Bochum, Germany; 5 Children’s Hospital, Marienhospital Wesel, 46483 Wesel, Germany

**Keywords:** cystic fibrosis, adults, health-related quality of life, Elexacaftor/Tezacaftor/Ivacaftor, longitudinal changes

## Abstract

Background: Longitudinal data on changes in health-related quality of life (HRQoL) in adult people with cystic fibrosis (pwCF) and the longitudinal effects of Elexacaftor/Tezacaftor/Ivacaftor therapy (ETI) on HRQoL or HRQoL domains are currently scarce. This study aimed to investigate the effects of ETI on HRQoL and compare them with those of pwCF who did not receive highly effective CFTR modulators over a longer period. Methods: Baseline assessment and follow-up data for 5.6 years in pwCF with (*n* = 21) and 6.5 years in pwCF without (*n* = 6) ETI (≥18 years) were evaluated. The assessment of HRQoL and clinical parameters was identical at both time points. HRQoL was assessed using the CFQ-R, and clinical outcomes included BMI, ppFEV1, and FEV1 z-score. Results: ETI was found to improve all HRQoL domains at more than four points over time, and their increases were significant except for vitality, digestion, treatment burden, and social functioning (*p* < 0.05). Without ETI, psychosocial domains remained almost constant, whereas most physical domains decreased over time. Conclusions: The results of the present study show that ETI therapy has a positive effect on HRQoL and clinical outcomes over time but not in pwCF without ETI treatment. Furthermore, our results suggest that disease progression over time affects the physical domains of HRQoL more than the psychosocial domains. Due to the small sample size and the heterogeneity of the study population (CFTR mutation genotype), the results should be interpreted with some caution.

## 1. Introduction

Cystic fibrosis (CF) is a chronic autosomal recessive genetic disease caused by a mutation in the gene responsible for the cystic fibrosis transmembrane conductance regulator gene (CFTR) [1]. This gene defect is responsible for respiratory manifestations, exocrine pancreatic insufficiency, malabsorption and malnutrition, CF-related diabetes mellitus (CFRD), reduced bone mass, osteoporosis, higher hospitalization rates, impaired quality of life, and reduced life expectancy [1,2]. However, reduced life expectancy and treatment burden affect health-related quality of life (HRQoL), and depression and anxiety are relatively common symptoms in CF [3,4].

Improved therapeutic approaches and the development of new drugs, such as CFTR modulators, have led to a steady increase in life expectancy [1]. CFTR modulators that target the underlying defect, such as the triple combination Elexacaftor/Tezacaftor/Ivacaftor (ETI), are available for approximately 90% of people with cystic fibrosis (pwCF) aged 12 years and older with at least one copy of the del508 mutation. PwCF treated with ETI improved lung function and nutritional status, had fewer exacerbations and respiratory symptoms, reduced sweat chloride concentrations, and improved quality of life [1,5,6,7].

In recent years, HRQoL has become an important endpoint in CF care [4,8,9]. To assess HRQoL in pwCF, the Cystic Fibrosis Questionnaire-Revised (CFQ-R) is commonly used to measure current disease-specific HRQoL and changes over time. The CFQ-R is a validated measure that includes general and CF-specific scales to assess the physiological and psychosocial aspects of HRQoL in CF [8,9].

There are few studies in the literature on long-term changes in HRQoL in adult pwCF. Over a period of 21 months, Dill et al. found no longitudinal differences in any of the physical HRQoL domains but did find individual differences in trends. On the contrary, significant trends were observed for the psychosocial HRQoL domains (emotional functioning, social functioning, and treatment burden). Significant predictors of psychosocial HRQoL over time were age, education, and ppFEV1 [10]. In cross-sectional studies, ppFEV1%, pulmonary exacerbations, anxiety and depression, and family, occupational, and social components were found to be independent predictors of HRQoL in CF [11,12].

Recent randomized controlled trials have shown that treatment with ETI leads to a significant improvement in the respiratory domain of the CFQ-R [1,5,6,7,13,14,15,16,17,18]. Only two studies evaluated the effect of ETI treatment on all 12 domains of the CFQ-R. Most of the 12 domains of the CFQ-R showed a significant improvement one to three months after the initiation of ETI treatment [16,18].

The aims of the present study were first to examine longitudinal changes in all domains of HRQoL with and without ETI treatment. In addition, changes over time in all HRQoL domains in the pwCF with and without ETI treatment were compared. For comparison, the HRQoL scores in the ETI group were recorded after several weeks of starting the ETI treatment and compared with a group that did not undergo ETI treatment. We hypothesize that HRQoL domain scores will change over time in both groups and that different changes will be observed depending on the type of medical treatment (with or without ETI).

## 2. Methods

### 2.1. Patients and Study Design

The present study is an amendment of the CFmobil project. The CFmobil project was a partially supervised physical activity intervention for pwCF aged ≥ 6 years, which has been described in detail elsewhere. The project was carried out between 2014 and 2018 at the Christine Herzog Centrum Ruhr (CHCR) and the participating hospitals (Ruhrlandklinik and Kinderklinik, Essen, Germany, and University Hospital for Paediatrics and Adolescent Medicine, Bochum, Germany) [19,20]. In the present study, pwCF aged ≥ 18 years from the University Hospital Essen (Ruhrlandklinik and Kinderklinik, Essen, Germany) and the University Hospital for Pediatrics and Adolescent Medicine (Bochum, Germany) agreed to continue participating. All 12 domains of the CFQ-R and clinical outcomes (weight, BMI, ppFEV1, and FEV1 z-scores) were compared with baseline values (T0) to examine long-term effects with and without ETI. Patients could not be randomized due to ETI therapy.

All participants had a confirmed diagnosis of CF based on two defining mutations in the CFTR gene. Written informed consent was obtained from all adult pwCF prior to enrollment. The study was approved by the Ethics Committees of the University Hospitals of Essen (14-6117-BO) and Bochum (15-53114) and is listed on ClinicalTrials (NCT03518697). Changes in HRQOL from baseline to follow-up were recorded and compared between a group that received ETI (ETI group) and a group that did not receive ETI treatment (non-ETI group). Baseline data collection (HRQoL and clinical parameters) was continuous with the enrolment of pwCF in the CFmobil project during the period of 2014 to mid-2018, with follow-up evaluation during the period of mid-2021 to mid-2022.

#### 2.1.1. Measurements

The assessments completed by the participants were identical to those performed at the beginning (T0) of the CFmobil project. Baseline data (T0) were collected between 2014 and 2018, and follow-up data (T5) were recorded in 2021–2022. Clinical parameters were recorded during the outpatient visit. The CFQ-R questionnaires were also completed by the pwCF at the end of the outpatient visit and returned to the CF team. The participation in the trial was not dependent on the duration of ETI therapy.

#### 2.1.2. Anthropometric Characteristics and Lung Function

Height was measured to the nearest 0.1 cm using a telescopic measuring rod (Seca 202; Seca, Hamburg, Germany). Body weight was assessed using an electronic flat scale (Seca 861; Seca, Hamburg, Germany), and body mass index (BMI) was calculated as weight divided by the square of height (kg/m^2^). The percent predicted Forced Expiratory Volume (ppFEV1) was determined according to the American Thoracic Society guidelines [21] using standard spirometric procedures (JAEGER MasterScreen Body; CareFusion, Höchberg, Germany) and expressed as a percentage of the predicted value. The Forced Expiratory Volume z-scores (FEV1-z-scores) were calculated using the Global Lung Initiative equations [22].

### 2.2. Health-Related Quality of Life

At baseline (T0) and follow-up (T5), all participants completed the CFQ-R questionnaire. The CFQ-R is a disease-specific questionnaire. It measures HRQoL in CF using 50 questions divided into 12 domains that provide information about the impact of the disease on the daily lives of children, adolescents, and adults pwCF and their parents or caregivers [9]. The 12 domains describe aspects of physical functioning, vitality, emotional functioning, social functioning, role, nutrition, treatment burden, health perception, body, weight, breathing, and digestion [8]. All domains are analyzed independently and standardized on a scale of 0–100. A higher score represents a higher quality of life in that domain. In the present study, all domains were used for comparison within and between groups to assess changes over time and to determine effects with and without ETI.

### 2.3. Statistics

All data are expressed as mean ± standard deviation (SD) with a 95% confidence interval (CI). Descriptive statistics were calculated, and data were tested for normal distribution (Shapiro–Wilk test). The outcomes used for the static analysis were changes from baseline (T0) to follow-up (T5) for ppFEV1, FEV1 z-cores, weight, BMI, and all 12 domains of the CFQ-R questionnaire. Changes over time were calculated using the Wilcoxon rank test. The ETI group and the non-ETI group were then analyzed separately for statistically significant changes over time for the CFQ-R domains and clinical outcomes. A Kruskal–Wallis test was used to compare the groups to identify significant differences between the different time points. Correlations in both groups between HRQoL domains scores, weight, BMI, ppFEV1, and FEV1z-score were evaluated using Spearman correlation coefficients. Statistical significance was set at 0.05 for all tests. Statistical analyses were performed using SPSS 28.0 (IBM Corp. Version 28.0. Armonk, NY, USA).

## 3. Results

### 3.1. Anthropometric Characteristics and Lung Function with and without ETI Therapy

A total of *n* = 28 pwCF were enrolled at follow-up (T5), with *n* = 21 pwCF receiving ETI treatment. The mean follow-up was 5.6 ± 0.8 years in the ETI group and 6.5 ± 0.5 years in the non- ETI group (*p* = 0.001). The mean duration of ETI treatment was 33 ± 25 weeks. The clinical results at baseline (T0) and at follow-up (T5) are shown in Table 1.

**Figure 1:**
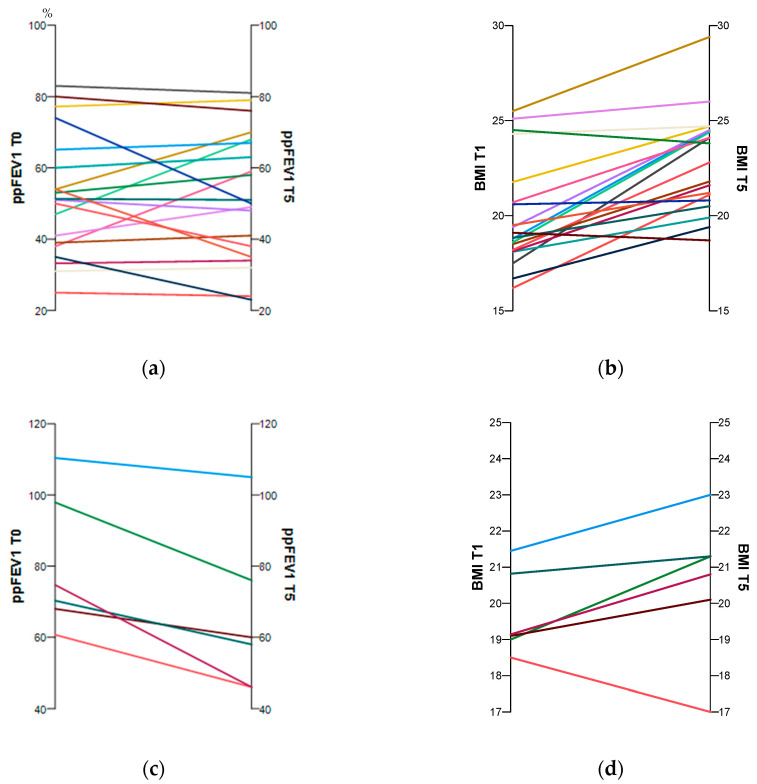
Individual changes in the subjects’ ppFEV1 and BMI between baseline and follow-up with (**a**,**b**) and without (**c**,**d**) CFTR treatment. The time trends for each pwCF are shown by the coloured lines.

At the test time follow-up (T5), participants in both groups were significantly older. There was a highly significant improvement in weight and BMI over time in the ETI group. The ppFEV1 changed only slightly, whereas the FEV1 z-score increased significantly. However, in the non-ETI group, weight and BMI remained stable over time. The ppFEV1 decreased significantly, as did the FEV1 z-score, but its change was not significant (Table 1). Figure 1a–d show the individual changes over time for ppFEV1 and BMI with and without CFTR treatment.

Significant between-group differences were found only for ppFEV1 and FEV1 z-score at baseline (T0). All other parameters were almost identical at baseline and follow-up (Table 1).

### 3.2. Health-Related Quality of Life

The HRQoL domains of the two groups over time are summarized in Table 2. In the HRQoL sub-scales of vitality, digestive, treatment burden, and social functioning, the ETI group showed modest improvements that were not significant over time. The other sub-domains of physical functioning, body image, eating health perceptions, respiratory, weight, emotional functioning, and role functioning improved significantly (Table 2, Figure 1).

In the non-ETI group, changes over time were less consistent in each of the sub-domains of HRQoL. In the psychosocial domains of treatment burden, emotional functioning, social functioning, and role functioning, a small, non-significant increase was found. However, in the subcategories of the physical domains, except for the domain of body image, a moderate but non-significant decrease was observed over time (Table 2).

**Figure 2:**
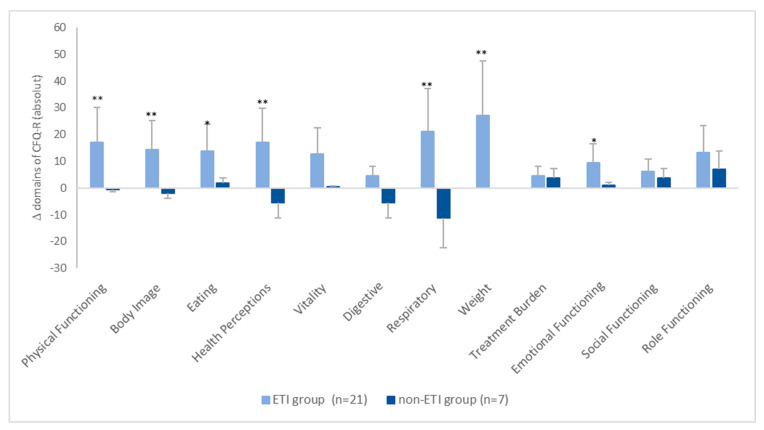
Mean absolute change in CFQ-R scores from baseline. * = *p* < 0.05, ** = *p* < 0.001.

The absolute changes in the CFQ-R scores between the two groups are shown in Figure 2. It can be seen that, in the ETI group, all scores increased by more than four points. However, in the non-ETI group, the changes in the individual domains ranged on average from −11.1 points (respiratory domain) to +6.9 points (role functioning) (Figure 2).

The comparison between the two groups at baseline and follow-up showed significant differences only for the sub-domains of respiratory and emotional functioning at baseline (T0). At follow up, no significant differences were found between the groups for either the physical or psychological domains (Table 2, Figure 1). However, it was found that the scores of each sub-domain of HRQoL were significantly lower in the ETI group than in the non-ETI group, except for the treatment burden domain.

### 3.3. Correlation of HRQoL Domains with Clinical Outcome Parameters at Baseline (T0) and Follow-Up (T5) and Their Changes over Time

The longitudinal associations and changes for HRQoL scores are shown in Table 3. The longitudinal changes between HRQoL domains and clinical outcomes in the non-ETI group showed a significant correlation for the digestive, body weight, and BMI domains (Table 3, while no significant correlations were found between the parameters in the ETI group (Table 3).

## 4. Discussion

The results of the present study show significant improvements in most HRQoL domains over a period of 5.6 years in pwCF treated with ETI for 33 ± 25 weeks. In contrast, in the non-ETI group, psychosocial domains changed little over 6.5 years, while the physical domains decreased, but not significantly.

Longitudinal assessment of HRQoL in adult pwCF has not been well-studied. This is somewhat surprising, as cross-sectional studies have shown that HRQoL monitoring provides additional information about disease-related limitations in pwCF in addition to routine assessments such as lung function measurement. The CFQ-R has been used as a validated measure that includes general and CF-specific scales to assess the physiological and psychosocial aspects of HRQoL in CF [8,9].

There are only a few studies in the literature on pwCF that examine the changes in HRQoL over time.

In the present study, we monitored HRQoL in adult pwCF over a period of 5.6 years in those treated with ETI for an average of 33 ± 25 weeks and over 6.5 years in the non-ETI group. This observation period significantly exceeds the duration of previously published studies, including the study by Dill et al., which had an observation period of only 23 months without ETI [10]. It also exceeds the study period of Castellanos et al., who examined the effect of ETI therapy on HRQoL over a period of 22 months [23]. However, it should be noted that although our study had a longer observation period, the average duration of ETI use in our pwCF was comparatively shorter than in the study by Castellanos et al. [23].

In contrast to our study with adult pwCF, HRQoL was determined in children and adolescents in the study by Castellanos et al. Respiratory domain scores were comparable at baseline but showed higher scores at follow-up. On the one hand, the greater changes could be due to the longer duration of treatment with ETI. On the other hand, the subjective perception of the positive change in the sensation of breathing (e.g., during exercise) may be more pronounced in children, which would explain the higher scores. However, this study did not report ppFEV1 values, so the comparability of the data is limited.

Clinical trials evaluating the efficacy of ETI treatment have shown significant improvements in weight, BMI, ppFEV1, and the respiratory component of HRQoL from four weeks to 26 months after treatment initiation. Improvement in ppFEV1 at the end of follow-up ranged from 10% to 13.8% and respiratory domain improvement from 16 to 20.2 points [1,5,6,7].

Consistent with these findings, our study also showed significant improvements in lung function, nutritional status, and HRQoL in individuals in the ETI group as a result of ETI therapy. It is interesting to note that there was also an increase in body weight and BMI in the group without ETI, indicating a better nutritional status. However, the increase in body weight is less than that in the ETI group. In the ETI group, the respiratory domain improved by 22.5 points, whereas the improvement at follow-up was only 2.31%.

The improvement in ppFEV1 was relatively small, which may be explained by the long period observed. However, the respiratory domain improved with ETI therapy to a similar extent as in other studies [1,5,6,7]. This could be because of negative affective responses such as breathlessness, coughing, dyspnoea-related anxiety, distress, and fear decrease during physical activities with ETI treatment [24]. This results in a reduction in the subjective negative sensation of breathing and an increase in the respiratory domain.

The HRQoL in pwCF is a multidimensional construct, including generic domains (physical functioning, vitality, health perceptions, emotional, social, and role functioning), and CF-specific domains (body image, eating disturbances, treatment burden, and respiratory and digestive symptoms) [3,13,25]. In the studies mentioned above, the assessment of HRQoL focused only on the respiratory domain. Other domains of HRQoL were not included. The respiratory domain, as a subset of HRQoL, only partially reflects the limitations of individual functioning in daily life in pwCF. This limits the information on changes in HRQoL in pwCF, and in particular, on the psychological effects of CFTR motility therapy.

Our study is one of the few to examine the impact of ETI therapy on all domains of HRQoL. In line with previous studies by Fajac et al. and DiMango et al. [16,17,18] on the effects of ETI treatment on HRQoL parameters, we also found similar improvements in all 12 domains of HRQoL in the ETI group by more than four points over time, especially in the physical domains of health perception, vitality, digestion, respiration, and weight. An improvement of more than four points is considered to be of clinical significance [26].

The ETI treatment in the current study was longer than in the published studies that examined all domains of HRQoL taking into account ETI therapy, with a mean duration of 33 weeks [16,17,18]. However, it should be noted that the observation period during which HRQoL was measured was 5.6 years. The length of time and the small groups studied make it difficult to compare the results of the different studies, as we recorded HRQoL at baseline and at follow-up, but not at the start of ETI treatment. We have not recorded ETI compliance in the ETI group. Treatment adherence influences well-being, thus no conclusions can be drawn about treatment adherence in terms of its influence on HRQoL.

The decrease in ppFEV1 in pwCF over time is affected by a number of factors. These factors include disease severity, microbial infections such as Pseudomonas aeruginosa, pancreatic insufficiency (PI), pancreatic failure (PS), and age and sex [27,28,29]. The decrease in ppFEV1 and pulmonary exacerbations in turn have a negative impact on the quality of life of pwCF [12,28,30]. In line with Dill et al. [10], we observed decreases in the physical domains in the non-ETI group. The pwCF examined in Dill’s study showed moderate disease severity, whereas our pwCF had a mild disease course at baseline.

However, we found a positive and significant correlation between health perception and ppFEV1 as well as the respiratory domain at follow-up. There was no significant correlation between changes over time in all clinical outcomes and HRQoL domains in the non-ETI group. Our results support previous findings that a decrease in ppFEV1 over time has a negative impact on different domains of HRQoL, regardless of disease severity.

However, we found a positive and significant correlation between health perception and ppFEV1 as well as the respiratory domain at follow-up. There was no significant correlation between changes over time in all clinical outcomes and HRQoL domains in the non-ETI group. It is likely that the decrease in pFEV1 increased psychological problems such as anxiety and depression, especially in the group without ETI [11,12]. This had a negative effect on HRQoL overall or on some of the domains of HRQoL. As we did not control for this, we cannot make precise statements about the frequency of anxiety and depression in the pwCF studied.

The present findings support that the physical aspects of HRQoL could have a stronger impact on HRQoL over time than the individual psychosocial aspects. This is probably due to the progression of the disease and the accompanying increasing limitations, as shown in a recent study [10]. The worsening of ppFEV1, pulmonary exacerbations, and disease severity progression in the non-ETI group of our study compared to the ETI group seems to have a greater impact on the physical aspects of HRQoL than on the psychosocial aspects.

Besides clinical factors, there are other important aspects that contribute to HRQoL. These include continuous CF care, family support, occupational and social components, and being well integrated into the social environment and having positive relationships [13,25]. The correlation coefficient between changes over time in HRQoL domains and clinical outcomes did not show a significant relationship in our study. It can be concluded that above listed factors had a positive impact on both the physical and psychosocial domains of the CFQ-R, leading to improvements in overall HRQoL. This could explain the relatively stable scores over time in the psychological domains, especially in the non-ETI group. However, the results of the present study should be interpreted with caution due to the small number and limited heterogeneity of the non-ETI group. In addition, the reduction in dyspnea and coughing with ETI treatment may lead to increased physical activity in daily life [31]. This suggests a positive effect on HRQoL as evidenced by improvements in vitality and other physical domains.

When discussing the results between the two studied groups, it should be noted that the non-ETI group has a significantly better ppFEV1 at baseline. It can be concluded that the non-ETI group has a milder course than the ETI group with a greater severity of disease at T0. Irrespective of the eligibility of pwCF for ETI therapy, differences in the severity of disease progression and other aspects of the disease (mutation classes and CFTR molecular phenotype) can be noted between the two groups, which may also have influenced HRQoL during the course of the observation period.

Besides the positive effects of ETI therapy on HRQoL, negative effects on some pwCFs are also reported. These include side effects (e.g., debilitating migraine and sound sensitivity), and also a fear of increase in other health complications or psychological problems (e.g., emotional highs and lows and loss of identity) [13,32]. We did not record these factors in the present study. Therefore, it cannot be ruled out that these factors also negatively influenced HRQoL in the studied pwCFs. When discussing the results of this longitudinal study, it is important to consider its limitations. The study investigated changes in HRQoL with and without ETI treatment over time. The results showed that most of the parameters assessed had positive effects with pwCF and ETI treatment. However, some parameters worsened or remained stable over time without ETI treatment. Despite these study strengths, the study had some limitations. Follow-up examinations were performed from 2021 to 2022 in the same way as the initial examinations (T0, baseline), and only data from patients older than 18 years at T0 were used for the present analysis. We performed the follow-up examinations at time T5 and not at the start of ETI treatment.

Therefore, no statements can be made about changes in quality of life over time before the initiation of ETI treatment. Only lung function ppFEV1 and FEV1 z-scores were recorded at this time point. Therefore, comparisons of our results with previously published studies on the effects of ETI therapy should be made with caution. The duration of ETI treatment in our studied pwCF varied from 13 to 118 weeks. This duration may have different impacts on HRQoL and clinical outcomes, and it cannot be disregarded that the effects may be greater with longer duration of treatment.

The clinical assessments at T5 occurred after the COVID-19 pandemic, and there were no pandemic-related restrictions. However, it is possible that there may be a small effect of individual social distancing restriction, leading to a reduction in social activities, on HRQoL.

In this study, pwCF were not randomly assigned to groups. The non-randomized allocation of groups to either ETI therapy or no ETI therapy may limit the interpretation of the results and conclusions.

The present study used a small group of pwCF without ETI therapy as a reference group. There were differences in mutation class and lung function at baseline, with greater disease severity in the ETI group. This limits the strength of the results, and larger reference groups should be studied in the future. However, this is challenging as only 10% of pwCF are not eligible for ETI therapy.

## 5. Conclusions

In conclusion, this longitudinal study highlights the efficacy and beneficial effects of ETI treatment on HRQoL and clinical parameters in a group of adult pwCF. Treatment with ETI improved HRQoL scores with a clinically relevant difference of more than four points. Without ETI treatment, disease progression and increased physical limitations have a greater impact on the physical domains of HRQoL. Good social integration and continuity of care appear to have a supportive effect and a positive impact on psychosocial aspects of HRQoL as people age. Due to the small sample size and the limited heterogeneity of the study population (CFTR mutation genotype), the results should be interpreted with some caution.

## Figures and Tables

**Table 1:** Demographic data and clinical outcome parameters of the ETI group and the non-ETI group over time.

	ETI Group (*n* = 21)				Non-ETI Group (*n* = 6)							
	Mean ± SD		95% CI Lower/Upper		Mean ± SD		95% CI Lower/Upper		between Groups *p*-Value		within Groups *p*-Value	
Variable	T0	T5	T0	T5	T0	T5	T0	T5	T0	T5	ETI Group	Non-ETI Group
Males (%)	67				67							
del F508 homozygous (in %)	60				67							
del F508 heterozygous (in %)	28				17							
Pancreatic insufficient (%)	82				100							
Pseudomonas aeruginosa (%)	54				33							
time between clinical assessments (yrs)	5.4 ± 0.8			5.0/5.8	6.5 ± 0.5		6.1/7.0		**0.001**			
duration of intake ETI (weeks)	33 ± 25											
Age (yrs)	25.9 ± 7.4	31.0 ± 7.6	22.5/29.2	27.5/34.4	26.3 ± 9.4	32.3 ± 8.7 †	17.6/35.0	24.2/40.4	0.640	0.796	**<0.001**	**0.016**
Heigth (cm)	171.2 ± 8.8		167.0/175.2		174.7 ± 11.0		164.6/184.9					
Weight (kg)	58.7 ± 11.6	67.1 ± 11.9	53.7/64.0	61.7/72.6	60.9 ± 7.3	62.3 ± 7.3	54.1/67.6	49.4/75.6	0.435	0.499	**<0.001**	0.207
BMI	19.9 ± 2.8	22.8 ± 2.6	18.77/21.2	21.6/23.9	19.9 ± 1.2	20.6 ± 1.7	18.8/21.0	18.9/22.3	0.405	0.101	**<0.001**	0.116
ppFEV1 (%pred)	51.9 ± 16.5	53.1 ± 17.9	44.4/59.5	45.0/61.3	78.6 ± 18.3	66.0 ± 20.6	61.6/95.5	46.9/85.1	**0.003**	0.249	0.578	**0.028**
FEV1 z-Score	−3.92 ± 1.3	−3.0 ± 1.9 †	−4.5/-3.4	−3.9/−2.1	−0.99 ± 1.62	−2.70 ± 1.68	−3.49/−0.5	−4.05/−1.19	**0.008**	0.568	**0.017**	0.128

T0 = assessment of habitual physical activity; clinical assessment and testing; T5 = follow-up clinical assessment and testing; SD = standard deviation, CI = 95% confidence intervals; between groups: Kruskal–Wallis test, within groups: Mann–Whitney U-test. † = significant differences (*p* < 0.05) between the two times measured within the group tested. Bold = significant group differences (*p* < 0.05) at both times measured.

**Table 2:** Descriptive Statistics for HRQoL scores over time with or without ETI treatment.

	ETI Group (*n* = 21)				Non-ETI Group (*n* = 6)							
	Mean ± SD		95% CI Lower/Upper		Mean ± SD		95% CI Lower/Upper		between Groups *p*-Value		within Groups *p*-Value	
	T0	T5	T0	T5	T0	T5	T0	T5	T0	T5	ETI Group	Non-ETI Group
**Physical** **domains**												
Physical Functioning	71.4 ± 19.5	89.2 ± 8.1	61.3/79.5	85.2/94.0	81.9 ± 13.6	78.0 ± 15.6	67.7/96.2	63.6/92.4	0.289	0.099	**0.002**	0.752
Body Image	56.1 ± 25.2	72.6 ± 24.6	46.3/69.3	66.1/86.7	70.4 ± 24.0	69.8 ± 25.4	45.2/95.6	46.3/93.4	0.239	0.852	**0.030**	0.463
Eating	86.8 ± 19.8	100.0 ± 0.0	76.7/95.5	100.0/100.0	87.0 ± 22.7	90.5 ± 17.5	63.2/110.8	74.3/106.6	0.712	0.408	**0.007**	0.180
Health Perceptions	51.9 ± 21.5	69.3 ± 16.0	40.5/60.5	30.1/77.5	68.5 ± 21.6	60.3 ± 25.6	45.9/91.1	36.7/83.9	0.175	0.383	**0.011**	0.345
Vitality	56.7 ± 18.6	69.6 ± 24.3	47.1/64.5	55.9/82.6	66.7 ± 23.0	61.9 ± 16.6	97.7/69.2	46.6/72.2	0.376	0.288	0.073	0.892
Digestive	82.0 ± 15.1	87.6 ± 11.7	74.5/88.9	81.9/94.5	87.0 ± 17.8	76.2 ± 20.7	68.4/105.7	57.0/95.4	0.408	0.234	0.096	0.684
Respiratory	62.4 ± 19.37	84.9 ± 15.6	53.2/71.8	76.5/93.6	79.6 ± 24.0	67.4 ± 21.4	54.4/104.8	47.7/87.2	**0.049**	0.087	**0.004**	0.225
Weight	61.7 ± 36.3	94.1 ± 13.1	43.4/79.4	86.6/100.0	83.3 ± 18.3	76.2 ± 25.2	64.5/102.5	52.9/99.5	0.242	0.114	**0.005**	0.498
**Psychosocial domains**												
Treatment Burden	70.4 ± 15.8	77.8 ± 16.7	62.4/77.6	68.1/86.1	63.0 ± 19.5	66.7 ± 12.8	42.5/83.4	54.8/78.6	0.408	0.147	0.068	0.463
Emotional Functioning	75.9 ± 16.8	87.1 ± 9.3	67.4/83.3	83.2/92.6	88.9 ± 21.4	89.5 ± 13.8	66.5/113.3	75.8/102.3	**0.026**	0.318	**0.046**	1.00
Social Functioning	68.5 ± 14.9	76.1 ± 15.4	61.2/83.0	70.1/85.4	74.1 ± 15.2	77.0 ± 19.1	58.1/90.0	59.3/94.6	0.512	0.852	0.246	0.463
Role Functioning	70.8 ± 24.5	91.2 ± 9.5	46.3/69.3	86.5/96.8	84.7 ± 13.4	89.3 ± 11.5	70.7/98.7	78.6/99.9	0.251	0.757	**0.012**	0.416

T0 = baseline assessment of HRQoL, T5 follow-up assessment of HRQoL, SD = standard deviation, CI = 95% confidence intervals; between groups: Kruskal–Wallis test; within groups: Mann-Whitney U-test. Bold = significant group differences (*p* < 0.05) at both times measured.

**Table 3:** Correlation coefficients between weight, BMI, lung function, and HRQoL scores of the CFQ-R in the ETI group (*n* = 21) and non-ETI group (*n* = 6) between baseline (T0) and follow-up (T5).

	ETI Group (*n* = 21)				Non-ETI Group (*n* = 6)			
	*p*-Value							
	Weight	BMI	ppFEV1	FEV z-Score	Weight	BMI	ppFEV1	FEV z-Score
**Physical domains**								
Physical Functioning	0.951	0.817	0.746	0.204	0.979	0.923	0.990	0.980
Body Image	0.974	0.746	0.988	0.326	0.666	0.535	0.541	0.382
Eating	0.166	0.087	0.870	0.951	**0.012**	**0.018**	0.960	0.839
Health Perceptions	0.528	0.504	0.689	0.635	0.668	0.849	0.380	0.542
Vitality	0.998	0.969	0.844	0.488	0.239	0.200	0.838	0.858
Digestive	0.282	0.165	0.111	0.222	0.578	0.509	0.878	0.566
Respiratory	0.931	0.846	0.442	0.631	0.791	0.721	0.721	0.802
Weight	0.960	0.681	0.479	0.593	0.736	0.695	0.574	0.366
**Psychosocial domains**								
Treatment Burden	0.414	0.287	0.636	0.434	0.880	0.882	0.778	0.651
Emotional Functioning	0.784	0.691	0.761	0.903	0.348	0.337	0.381	0.310
Social Functioning	0.185	0.108	0.345	0.883	0.959	0.913	0.391	0.358
Role Functioning	0.988	0.785	0.887	0.134	0.836	0.912	0.970	0.864

Spearman rank correlation coefficients were used to test the relationship between parameters. Significant results (*p* < 0.05) are shown in bold.

## Data Availability

Data sharing is not applicable.

## References

[1] Shteinberg M., Haq I.J., Polineni D., Davies J.C. (2021). Cystic fibrosis. Lancet.

[2] Dickinson K.M., Collaco J.M. (2021). Cystic Fibrosis. Pediatr. Rev..

[3] Quittner A.L., Saez-Flores E., Barton J.D. (2016). The psychological burden of cystic fibrosis. Curr. Opin. Pulm. Med..

[4] Quittner A.L., Goldbeck L., Abbott J., Duff A., Lambrecht P., Solé A., Tibosch M.M., Brucefors A.B., Yüksel H., Catastini P. (2014). Prevalence of depression and anxiety in patients with cystic fibrosis and parent caregivers: Results of The International Depression Epidemiological Study across nine countries. Thorax.

[5] Sutharsan S., McKone E.F., Downey D.G., Duckers J., MacGregor G., Tullis E., Van Braeckel E., Wainwright C.E., Watson D., Ahluwalia N. (2022). Efficacy and safety of elexacaftor plus tezacaftor plus ivacaftor versus tezacaftor plus ivacaftor in people with cystic fibrosis homozygous for F508del-CFTR: A 24-week, multicentre, randomised, double-blind, active-controlled, phase 3b trial. Lancet Respir. Med..

[6] Middleton P.G., Mall M.A., Dřevínek P., Lands L.C., McKone E.F., Polineni D., Ramsey B.W., Taylor-Cousar J.L., Tullis E., Vermeulen F. (2019). Elexacaftor–Tezacaftor–Ivacaftor for Cystic Fibrosis with a Single Phe508del Allele. N. Engl. J. Med..

[7] Heijerman H.G.M., McKone E.F., Downey D.G., Van Braeckel E., Rowe S.M., Tullis E., Mall M.A., Welter J.J., Ramsey B.W., McKee C.M. (2019). Efficacy and safety of the elexacaftor plus tezacaftor plus ivacaftor combination regimen in people with cystic fibrosis homozygous for the F508del mutation: A double-blind, randomised, phase 3 trial. Lancet.

[8] Quittner A.L. (1998). Measurement of quality of life in cystic fibrosis. Curr. Opin. Pulm. Med..

[9] Quittner A.L., Buu A., Messer M.A., Modi A.C., Watrous M. (2005). Development and Validation of the Cystic Fibrosis Questionnaire in the United States. Chest.

[10] Dill E.J., Dawson R., Sellers D.E., Robinson W.M., Sawicki G.S. (2013). Longitudinal Trends in Health-Related Quality of Life in Adults with Cystic Fibrosis. Chest.

[11] Ancel J., Launois C., Perotin J.-M., Ravoninjatovo B., Mulette P., Hagenburg J., Malet J., Griffon M., Carré S., Lebargy F. (2022). Health-Related Quality of Life in Adults with Cystic Fibrosis: Familial, Occupational, Social, and Mental Health Predictors. Healthcare.

[12] Cronly J.A., Duff A.J., A Riekert K., Fitzgerald A.P., Perry I.J., A Lehane E., Horgan A., A Howe B., Ni Chroinin M., Savage E. (2019). Health-Related Quality of Life in Adolescents and Adults with Cystic Fibrosis: Physical and Mental Health Predictors. Respir. Care.

[13] Aspinall S.A., Mackintosh K.A., Hill D.M., Cope B., McNarry M.A. (2022). Evaluating the Effect of Kaftrio on Perspectives of Health and Wellbeing in Individuals with Cystic Fibrosis. Int. J. Environ. Res. Public Health.

[14] Nichols D.P., Paynter A.C., Heltshe S.L., Donaldson S.H., Frederick C.A., Freedman S.D., Gelfond D., Hoffman L.R., Kelly A., Narkewicz M.R. (2022). Clinical Effectiveness of Elexacaftor/Tezacaftor/Ivacaftor in People with Cystic Fibrosis: A Clinical Trial. Am. J. Respir. Crit. Care Med..

[15] Carnovale V., Iacotucci P., Terlizzi V., Colangelo C., Medio P., Ferrillo L., De Gregorio F., Francalanci M., Taccetti G., Buonaurio S. (2021). Effectiveness and safety of elexacaftor/tezacaftor/ivacaftor in patients with cystic fibrosis and advanced lung disease with the Phe508del/minimal function genotype. Respir. Med..

[16] DiMango E., Spielman D.B., Overdevest J., Keating C., Francis S.F., Dansky D., Gudis D.A. (2021). Effect of highly effective modulator therapy on quality of life in adults with cystic fibrosis. Int. Forum Allergy Rhinol..

[17] Ejiofor L.C.K., Mathiesen I.H.M., Jensen-Fangel S., Olesen H.V., Skov M., Philipsen L.K.D., Pedersen C.L., Pressler T. (2020). Patients with cystic fibrosis and advanced lung disease benefit from lumacaftor/ivacaftor treatment. Pediatr. Pulmonol..

[18] Fajac I., Daines C., Durieu I., Goralski J.L., Heijerman H., Knoop C., Majoor C., Bruinsma B.G., Moskowitz S., Prieto-Centurion V. (2022). Non-respiratory health-related quality of life in people with cystic fibrosis receiving elexacaftor/tezacaftor/ivacaftor. J. Cyst. Fibros..

[19] Welsner M., Gruber W., Mellies U., Olivier M., Sutharsan S., Taube C., Dillenhoefer S., Koerner-Rettberg C., Stehling F. (2021). Trainability of Health-Related and Motor Performance Fitness in Adults with Cystic Fibrosis within a 12-Month Partially Supervised Exercise Program. Pulm. Med..

[20] Gruber W., Stehling F., Blosch C., Dillenhoefer S., Olivier M., Koerner-Rettberg C., Sutharsan S., Mellies U., Taube C., Welsner M. (2022). Effects of a Long-Term Monitored Exercise Program on Aerobic Fitness in a Small Group of Children with Cystic Fibrosis. Int. J. Environ. Res. Public Health.

[21] Miller M.R., Hankinson J., Brusasco V., Burgos F., Casaburi R., Coates A., Crapo R., Enright P., Van Der Grinten C.P.M., Gustafsson P. (2005). Standardisation of spirometry. Eur. Respir. J..

[22] Quanjer P.H., Stanojevic S., Cole T.J., Baur X., Hall G.L., Culver B.H., Enright P.L., Hankinson J.L., Ip M.S.M., Zheng J. (2012). Multi-ethnic reference values for spirometry for the 3–95-yr age range: The global lung function 2012 equations. Eur. Respir. J..

[23] Castellanos C.X., Osterbauer B., Hasday S., Keens T.G., Koempel J., Ference E.H. (2022). Improvement in sinonasal quality-of-life indicators for pediatric patients with cystic fibrosis treated with elexacaftor-tezacaftor-ivacaftor. Int. Forum Allergy Rhinol..

[24] Carrieri-Kohlman V., Donesky-Cuenco D., Park S.K., Mackin L., Nguyen H.Q., Paul S.M. (2010). Additional evidence for the affective dimension of dyspnea in patients with COPD. Res. Nurs. Health.

[25] Keyte R., Kauser S., Mantzios M., Egan H. (2022). The psychological implications and health risks of cystic fibrosis pre- and post- CFTR modulator therapy. Chronic Illn..

[26] Quittner A.L., Modi A.C., Wainwright C., Otto K., Kirihara J., Montgomery A.B. (2009). Determination of the Minimal Clinically Important Difference Scores for the Cystic Fibrosis Questionnaire-Revised Respiratory Symptom Scale in Two Populations of Patients with Cystic Fibrosis and Chronic Pseudomonas aeruginosa Airway Infection. Chest.

[27] Adler F.R., Liou T.G. (2016). The Dynamics of Disease Progression in Cystic Fibrosis. PLoS ONE.

[28] Habib A.-R.R., Manji J., Wilcox P.G., Javer A.R., Buxton J.A., Quon B.S. (2015). A Systematic Review of Factors Associated with Health-Related Quality of Life in Adolescents and Adults with Cystic Fibrosis. Ann. Am. Thorac. Soc..

[29] Caley L., Smith L., White H., Peckham D. (2021). Average rate of lung function decline in adults with cystic fibrosis in the United Kingdom: Data from the UK CF registry. J. Cyst. Fibros..

[30] Flume P.A., Suthoff E.D., Kosinski M., Marigowda G., Quittner A.L. (2019). Measuring recovery in health-related quality of life during and after pulmonary exacerbations in patients with cystic fibrosis. J. Cyst. Fibros..

[31] Zaher A., ElSaygh J., Elsori D., ElSaygh H., Sanni A. (2021). A Review of Trikafta: Triple Cystic Fibrosis Transmembrane Conductance Regulator (CFTR) Modulator Therapy. Cureus.

[32] Purkayastha D., Agtarap K., Wong K., Pereira O., Co J., Pakhale S., Kanji S. (2023). Drug-drug interactions with CFTR modulator therapy in cystic fibrosis: Focus on Trikafta®/Kaftrio®. J. Cyst. Fibros..

